# Effectiveness and safety of ICIs for the treatment of advanced CC: a systematic review and meta-analysis

**DOI:** 10.3389/fimmu.2025.1542850

**Published:** 2025-03-11

**Authors:** Nurbia Ibibulla, Pengfei Lu, Yiliyaer Nuerrula, Xueqin Hu, Mulati Aihemaiti, Yubo Wang, Hua Zhang

**Affiliations:** ^1^ Cancer Center, The First Affiliated Hospital of Xinjiang Medical University, Xinjiang Uygur Autonomous Region, Urumqi, China; ^2^ Pharmacy Department, The First Affiliated Hospital of Xinjiang Medical University, Xinjiang Uygur Autonomous Region, Urumqi, China

**Keywords:** cervical cancer, immunotherapy, checkpoint inhibitors, ICIS, effectiveness

## Abstract

**Background:**

The use of immune checkpoint inhibitors has recently become a promising and innovative therapeutic option for patients suffering from advanced recurrent or metastatic cervical cancer(CC), and several studies of immunotherapy have been published or have revealed stage-by-stage results at international congresses. Nevertheless, there is a lack of meta-analyses of ICIs for advanced CC in past Meta-analysis.

**Method:**

This meta-analysis rigorously followed the PRISMA guidelines, using Review Manager V.5.4 and R(v4.2.2) software for data synthesis. Hazard ratios, risk ratios, and risk differences were calculated, with statistical significance assessed via the Mantel-Haenszel test. Heterogeneity was evaluated using the Higgins I^2^ statistic, and sensitivity analyses were conducted if heterogeneity surpassed 50%. The efficacy outcomes examined and gathered included the overall response rate (ORR), progress-free survival, overall survival(OS), and the adverse events (AEs), crucial for understanding the efficacy and safety of ICIs in advanced cervical cancer.

**Result:**

The results demonstrate significant efficacy and manageable safety of ICIs in advanced cervical cancer. In RCTs, ICIs improved OS (HR = 0.66, 95% CI: 0.58-0.75, *P* < 0.00001) and PFS (HR = 0.67, 95% CI: 0.59-0.75, *P* < 0.0001), with a 34% and 33% reduction in death and progression risks, respectively. ORR was higher in ICIs groups (RR = 1.39, 95% CI: 1.08-1.80, *P* = 0.01). Single-arm studies supported these findings (ORR: RD = 0.31, 95% CI: 0.22-0.40, *P* < 0.0001). Safety profiles were manageable, with comparable TRAEs in RCTs and higher incidences in single-arm studies. Subgroup analysis revealed superior OS benefits in PD-L1-positive patients (CPS ≥1, HR = 0.65, 95% CI: 0.50–0.84, *P* = 0.001) and significant efficacy in squamous cell carcinoma (HR = 0.67, *P* < 0.00001). Sensitivity analysis confirmed robust OS results (I² = 0%) and stable ORR despite heterogeneity. Risk of bias was low to moderate.

**Conclusion:**

Our meta-analysis reveals that immune checkpoint inhibitors (ICIs) significantly prolong overall survival in advanced cervical cancer patients, reducing the hazard ratio for death. Despite heterogeneity in outcomes, ICIs offer substantial treatment benefits. Further research is needed to optimize usage and monitor AEs.

**Systematic Review Registration:**

https://www.crd.york.ac.uk/PROSPERO, identifier CRD42023387789.

## Introduction

CC ranks as the fourth most common cancer and the fourth leading cause of cancer-related deaths in women worldwide. Recent projections indicate that CC will contribute to approximately 342,000 fatalities worldwide in 2020, comprising nearly 30% of all female cancer deaths. Disturbingly, a staggering 90% of these cases and deaths occur in low and middle-income countries, underscoring the disparities in access to healthcare and outcomes (1, 2). Disparities in five-year OS rates are evident across stages, with early-stage disease exhibiting an OS of approximately 92%, locally advanced stages at 65%, and a stark reduction to 17% for metastatic cases (3). The clinical outlook for patients with recurrent or metastatic (r/m) CC is notably poor, as shown by an estimated OS of 5 to 16 months and median progression-free survival (PFS) of only 2 to 5 months (4–6) additionally, approximately 6% of women diagnosed with CC present with primary metastatic disease. The poor prognosis is mainly attributed to the scarcity of treatment options, although most patients experiencing metastatic or recurrent stages manage to gain some advantages from systemic treatments, such as chemotherapy (CT) with or without angiogenesis inhibitors and immunotherapy (7). However, therapeutic avenues for advanced CC beyond first-line CT combined with bevacizumab are scarce. The response rate for second-line chemotherapy monotherapy in r/m CC is a mere 15%-20%, and the median survival duration fails to exceed two years (8). Consequently, there is a pressing imperative to discover and develop more efficacious novel interventions to adequately address the therapeutic requirements of patients grappling with advanced CC.

Progress in medical science and technology has greatly enhanced our understanding of cervical carcinogenesis, especially emphasizing the critical role of ongoing infections with high-risk human papilloma-virus (HPV) strains as the primary cause in most cases of CC (9–11). The pathogenesis involves HPV-positive cells subverting the host immune defenses by suppressing acute inflammatory responses and immune recognition mechanisms (12, 13). Emerging research suggests that this interplay between viral oncogenesis and host inflammatory pathways may potentiate the induction of immune checkpoint blockade therapy, notably the programmed death-ligand 1 (PD-L1) inhibitors. This therapeutic modality has garnered substantial attention for its potential in treating HPV-related CC and is increasingly being recognized as a frontrunner in the oncological management of the disease. Despite the promising results exhibited by numerous clinical and preclinical evaluations, to date, pembrolizumab remains the sole PD-L1 inhibitor granted approval for clinical application in this context.

Recent breakthroughs in CC research have illuminated the potential of immune checkpoint inhibitors (ICIs) as a viable treatment option for patients with advanced disease. The introduction of ICIs into CC treatment strategies has sparked intense research interest, particularly within the past few years, as evidenced by the proliferation of clinical trials investigating their effectiveness across different stages of the disease, from advanced to recurrent cases (14). ICIs work by augmenting T cell-mediated cytotoxicity against cancer cells, leading to encouraging treatment responses and improved survival outcomes in patients with recurrent and metastatic CC. A systematic review and meta-analysis were undertaken to critically appraise the data from the most recent clinical trials investigating ICIs in the treatment of CC. This comprehensive analysis includes data from trials with approved ICI indications, as well as those exploring ICI monotherapy and combination therapy paradigms, in addition to studies that are currently underway. The primary aim of this study was to evaluate the potential of ICIs as innovative therapeutic agents, with a particular focus on their ability to address a critical gap in the treatment landscape of advanced or recurrent CC.

## Materials and methods

The protocol for our systematic review has been formally registered with PROSPERO, assigned the unique identifier CRD42023387789. Adhering strictly to the PRISMA guidelines (15), we have structured our reporting approach to ensure clarity, transparency, and reproducibility. This approach ensures that our review meets the highest standards of systematic review methodology.

### Search strategy

Three researchers (Nurbia I, Hu X, and Mulati A) conducted independent searches in various databases, including PubMed, EMBASE, MEDLINE, Web of Science, and the Cochrane Library, as well as scanning meeting abstracts from the American Society of Clinical Oncology (ASCO), the European Society for Medical Oncology (ESMO), and the Society of Gynecological Oncology (SGO) to uncover unpublished studies. Guided by the PICOS framework (**Table 1**), the search strategies were tailored accordingly. Our search covered the period from 1st January 2017 to 31st January 2024, and the detailed search strategy can be found in **Appendix A** of the **Supplementary Materials**.

**Table 1:** Criteria for study inclusion and exclusion in the systematic search design.

Component	Inclusion criteria	Exclusion criteria
patients/participants	Female patients (at least 18 years old) with histologically or cytologically proven advanced cervical cancer regardless of the subtype (include persistent, recurrent, or metastatic CC)	• Early-stage cervical cancer • Only locally advanced cervical cancer • Undiagnosed cervical cancer • Other cancers
Intervention & Comparator	Intervention: Immune checkpoint inhibitors therapy (monotherapy or combination) Comparison: Any (including chemotherapy, targeted therapy, surgery or placebo) or no comparison	No immune checkpoint inhibitors on the intervention
Outcome	Study reported at least one measure of survival and safety outcomes: overall survival (OS)/objective response rate (ORR) and adverse events (AE)	• Unspecified/not clearly outcomes relating to intervention. • Failure to meet outcome indicators/not reported.
Language	English	Other languages not translated into English.
Publication	Complete text of the article	• Conference abstract, letter to editors, news, analysis, and editorials, etc. • complete text not available, out of topic or no clinical endpoints.

### Inclusion and exclusion criteria

Studies were included if they (i)Female patients (≥18 years) with histologically or cytologically proven advanced CC, regardless of whether they are treated with ICIs alone or in combination, are eligible for inclusion; (ii)They fulfill the PICO inclusion criteria; (iii) The lesions can be measured according to RECIST v1.1 (16, 17); (iv) include phase I-IV clinical trials that provide information on the safety and effectiveness of ICIs (monotherapy or combination) in patients with advanced CC that have recurred or metastasized; (v) Despite the acknowledged potential for higher bias in such studies, single-arm trials were included in this systematic review, given that they are commonly used in phase I and II clinical trials in oncology, and the scarcity of comparative studies in this emerging field. Nevertheless, the results should be approached with caution. Exclusions were made for studies that were (i) meta-analyses, reviews, case reports, correspondences, personal opinions, or studies involving *in vitro* or animal models, among others; (ii) complete text not available, out of topic, or no clinical endpoints (**Table 1**).

### Data extraction and strategy for data synthesis

Documentation retrieval, research selection, data extraction, and risk of bias assessment were carried out independently by two authors (Nurbia I, Hu X), and discrepancies were reviewed by another author (Mulati A). The extracted data was categorized into four primary sections: (i) study characteristics, encompassing the author, year of publication, country of origin, and study phase; (ii) target population details, including the number of patients; (iii) specific clinical factors including PD-L1 expression status, pathological classification, and the therapeutic agents used; (iv) Key results concerning primary and secondary outcomes. For randomized controlled trials (RCTs), the primary endpoints were overall survival (OS) and progression-free survival (PFS). For single-arm studies, the primary endpoint was the objective response rate (ORR). Additionally, we focused on secondary outcomes, including disease control rate (DCR), duration of response (DOR), and a detailed evaluation of treatment-related adverse events (AEs), noting their frequency and severity grades (18). The results were summarized narratively and presented graphically in **Supplementary Tables S1** and **S2**.

The extracted data was comprehensively analyzed. For dichotomous data, we used the hazard ratio (HR), relative risk (RR) and risk difference (RD) as the main indicators and calculated the respective 95% confidence intervals. To measure statistical heterogeneity, we utilized the *I* ^2^ statistic, considering values above 50% as significant indicators of heterogeneity. Given the detected heterogeneity, we chose the random effects model (REM) as our principal method of analysis. Sensitivity analyses were performed using R(v4.2.2) software to further scrutinize the sources of heterogeneity, while all other analyses were conducted using Review Manager V.5.4 (Rev Man 5.4) (19). Subgroup analyses were also performed to identify potential biases or methodological disparities among the included trials. The combined effect size was plotted using a forest plot.

### Quality assessment

The evaluation of potential bias and research quality was conducted independently by two reviewers (Nurbia I and Hu X), with any discrepancies resolved through discussion with a third reviewer (Mulati A). For randomized control trials (RCTs), we utilized the updated Cochrane Risk of Bias tool for randomized trials (RoB-2) to assess the risk of bias (20, 21). Non-randomized studies of intervention effects are essential for evaluating healthcare, particularly in phase I and II trials related to cancer. However, the tools currently available for comparative trials are inadequate for non-comparative studies. There is a lack of a universally accepted tool for assessing the risk of bias in such studies. To bridge this deficiency, the Non-Randomized Studies of Interventions tool (ROBINS-I) has been introduced to assess the risk of bias in single-arm cohort trials, providing a crucial asset for systematic reviews incorporating non-randomized studies (22). In cases where studies lacked a proper efficacy evaluation, the risk of bias in the clinical safety assessment was considered. To confirm the reliability of our results, a sensitivity analysis will be conducted that incorporates the outcomes of the bias assessment.

## Results

### Study selection

**Figure 1:**
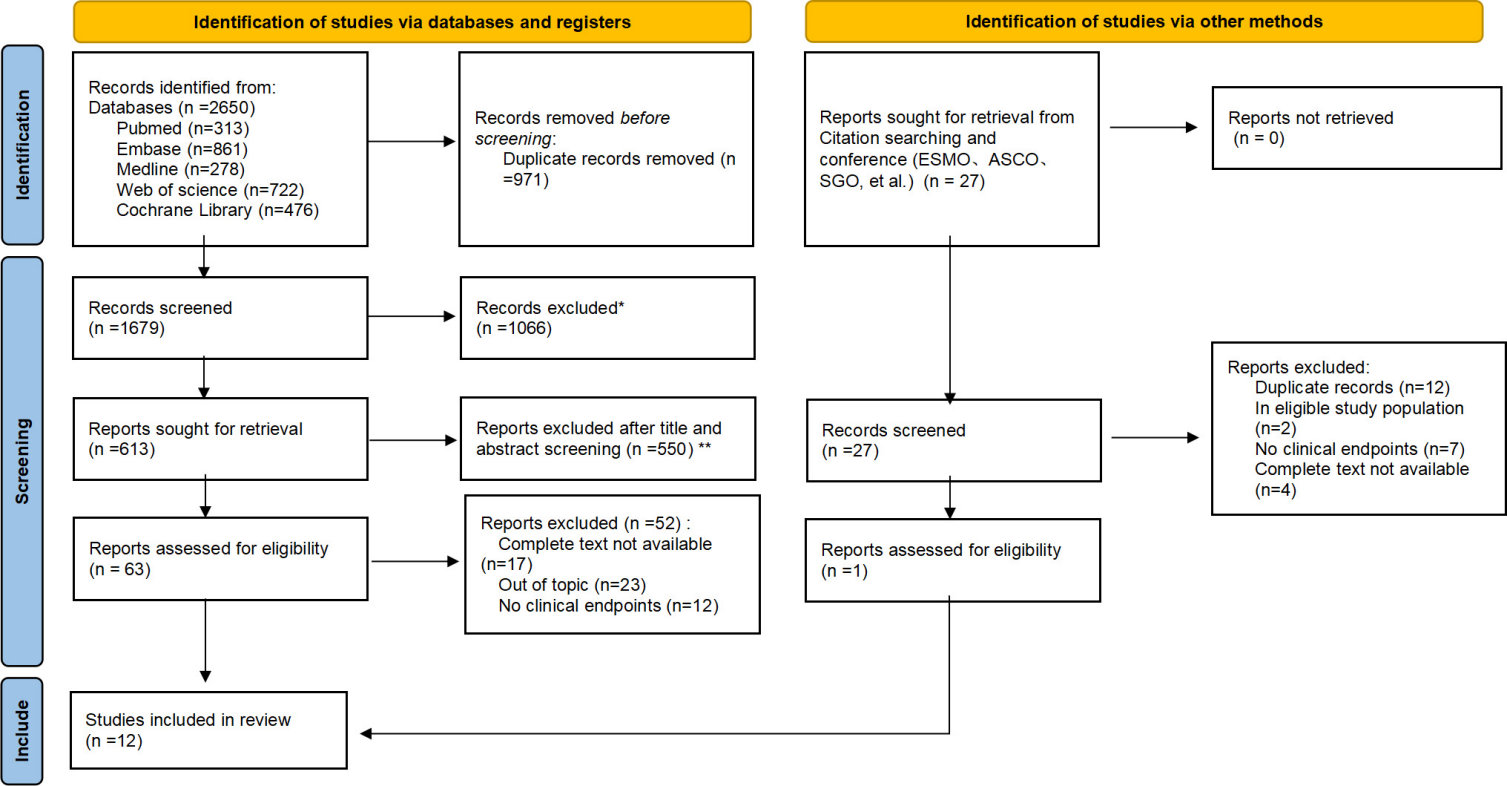
PRISMA flowchart of the screening and inclusion process. *Records excluded: Case reports (n=71); Conference abstract (n=289); Editorial (n=64); News (n=23); Note (n=27); Reviews (n=378); Correspondence, letters and personal opinions (n=214). *Reports excluded after title and abstract screening (n=550): No cervical cancer (n=110); No immunotherapy (n=91); Retrospective (n=215); Updated/more detailed report of same trial available/included (n=134).

The PRISMA flowchart, depicted in **Figure 1**, clearly outlines the systematic approach we adopted for our analysis. Out of the 2650 studies found by the electronic database searches, 63 were fully analyzed. Ultimately, 12 publications were selected using the PRISMA method of article selection for this review (according PRISMA checklist).

### Study characteristic

In our analysis, we reviewed 12 clinical studies on immunotherapies for recurrent or metastatic cervical cancer, comprising three RCTs (23–25) and nine single-arm studies (26–34). These studies included patients with a median age of 42–69 years, measurable disease, and a performance status score of 0 or 1. The studies evaluated various agents and combination therapies targeting immune checkpoints and pathways. Specifically, PD-1 inhibitors (pembrolizumab, camrelizumab, sintilimab, zimberelimab) and PD-L1 inhibitors (cemiplimab, atezolizumab) were investigated, along with CTLA-4 inhibitor ipilimumab. Innovative combinations included GX-188E plus pembrolizumab, balstilimab plus zalifrelimab, tisotumab vedotin, camrelizumab plus apatinib, and chemotherapy combined with immunotherapies. Detailed study characteristics and patient baseline data are provided in the supplementary materials (**Supplementary Tables S1**, **S2**).

### Overview of the evaluated effectiveness and safety profiles

The safety and survival of advanced CC patients undergoing immunotherapy were validated by our comprehensive review. The differences in trial designs, treatments, and their combinations, along with varied selection criteria, played a major role in the significant heterogeneity seen in the outcomes. The results were found to fluctuate over a wide range: 10 trials reported OS, which varied from 8.5 to 32.1 months, with two indicating no data (median OS not reached or not reported). ORR varied widely from 12.2% to 84%. Specifically, in trials using dual ICIs, an ORR of 25.6% was achieved, whereas trials using ICIs in combination with tyrosine kinase inhibitors (TKIs) reported an ORR of 55.6%. Among PD-L1-positive patients, ORR ranged from 11% to 69.6%, while in PD-L1-negative groups, ORR ranged from 9.1% to 50%. The safety of the interventions was assessed through treatment-related adverse events (TRAEs) or AEs, graded using the National Cancer Institute’s Common Terminology Criteria for Adverse Events (CTCAE) version 4.03. These TRAEs, specific to each treatment combination, were identified by the investigators. Occurring in 24.5% to 100% of patients treated with ICIs, irrespective of attribution, TRAEs included Grade 3 adverse events, which the CTCAE categorizes as severe or medically significant, though not immediately life-threatening (35). The safety assessments of the interventions were conducted through TRAEs, with AEs of grade 3 or higher occurring in 11%-79% of patients within the ICI groups. The most commonly reported adverse events (AEs) of any severity involved reactions of the hematologic and gastrointestinal systems, such as anemia, diarrhea, nausea, vomiting, and constipation. Additionally, other adverse events reported included fatigue, alopecia, hypothyroidism, neutropenia, and elevations in AST/ALT.

### Effectiveness

**Figure 2:**
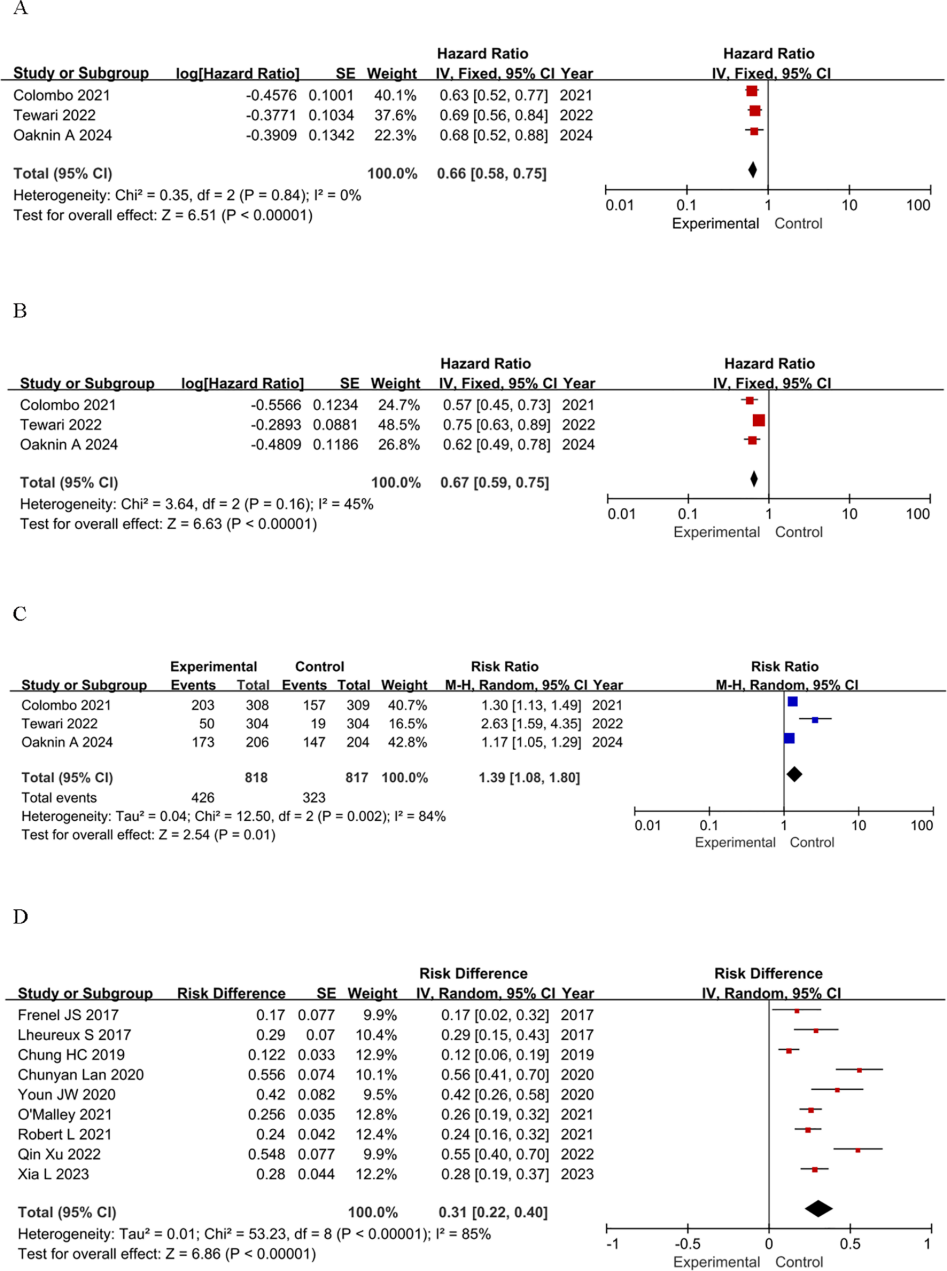
Forest plots of effectiveness outcomes for the included studies: **(A)** OS in RCTs; **(B)** PFS in RCTs; **(C)** ORR in RCTs; **(D)** ORR in single-arm studies.

The main endpoints of focus in the chosen studies were OS and PFS in RCTs and ORR in single-arm clinical trials. A statistically significant HR for OS was observed in the RCTs, indicating a favorable outcome for patients treated with ICIs. Notably, the OS in the ICIs group was significantly longer than that of the control group, with a pooled HR for death of 0.66 (95% confidence interval [CI]: 0.58-0.75, *P <* 0.00001). This finding indicates a 34% reduction in the risk of death compared to the control groups (**Figure 2A**). For PFS, patients receiving ICIs also showed a statistically significant benefit, with a pooled HR for progression or death of 0.67 (95% CI: 0.59-0.75, *P* < 0.0001), suggesting a 33% reduction in the risk of disease progression or death (**Figure 2B**). In terms of ORR, the RR in the ICIs group was 1.39 (95% CI: 1.08-1.80, *P*=0.01), which was significantly higher than that of the control group (**Figure 2C**). In single-arm studies, the pooled RD was 0.31 (95% CI: 0.22-0.40, *P* < 0.0001), indicating a promising ORR across various groups (**Figure 2D**). These findings highlight the potential efficacy of ICIs in advanced cervical cancer.

### Safety

**Figure 3:**
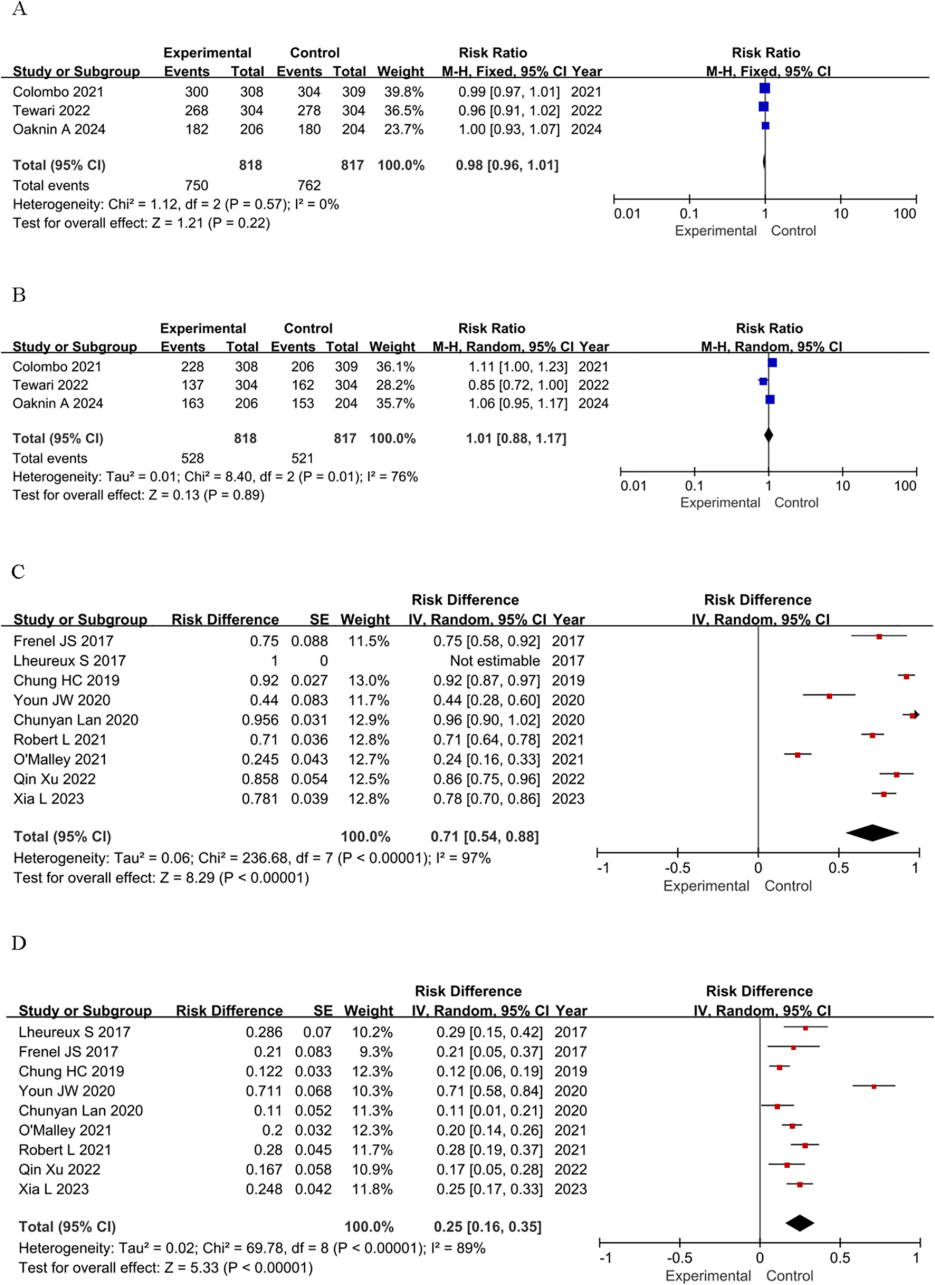
Forest plots of safety outcomes stratified by adverse events: **(A)** Adverse Events of Any Grade in RCTs; **(B)** ≥G3 AEs in RCTs; **(C)** Adverse Events of Any Grade in single-arm studies; **(D)** ≥G3 AEs in single-arm studies.

To assess the safety of immunotherapy, we further analyzed the immune-mediated AEs regardless of attribution. The assessment of TRAEs encompassed two primary aspects: the total count of adverse events attributed to treatment, as well as the frequency of those AEs that were graded above or equal to grade 3 (≥G3). Utilizing the CTCAE standard, G3 AEs were classified as severe or medically significant, yet not posing an imminent threat to life (36). We used RR, RD, and 95% credibility intervals as summary statistics to quantify the safety of ICIs. The safety profile of immunotherapy is manageable both in single-agent and RCTs. The incidence of any-grade TRAEs in RCTs is nearly identical to that in the control group (RR = 0.98, 95% CI: 0.96–1.01, *P* = 0.22), and the incidence of severe adverse events (≥G3) is also comparable (RR = 1.01, 95% CI: 0.88–1.17, P = 0.89) (**Figures 3A, B**). In single-arm studies, the incidence of any-grade TRAEs is high (RD = 0.71, 95% CI: 0.54–0.88), and the incidence of severe adverse events (≥G3) is moderate (RD = 0.25, 95% CI: 0.16–0.35) (**Figures 3C, D**). In conclusion, the safety profile of immunotherapy is manageable in both single-agent and RCTs, but continued vigilance in monitoring and managing adverse events is essential.

### Subgroup analysis

A subgroup analysis was conducted, focusing on the expression patterns of PD-L1 and the histologic characteristics of the samples (37–40). Studies not reporting relevant factors were excluded. Given the potential impact of PD-L1 expression on patient survival, we generated forest plots that illustrate the HR and RD across various PD-L1 expression statuses. Notably, most of the studies we incorporated utilized the CPS as a metric, which is calculated by dividing the number of PD-L1-positive cells by the total viable tumor cells and multiplying by 100 (41, 42).

**Figure 4:**
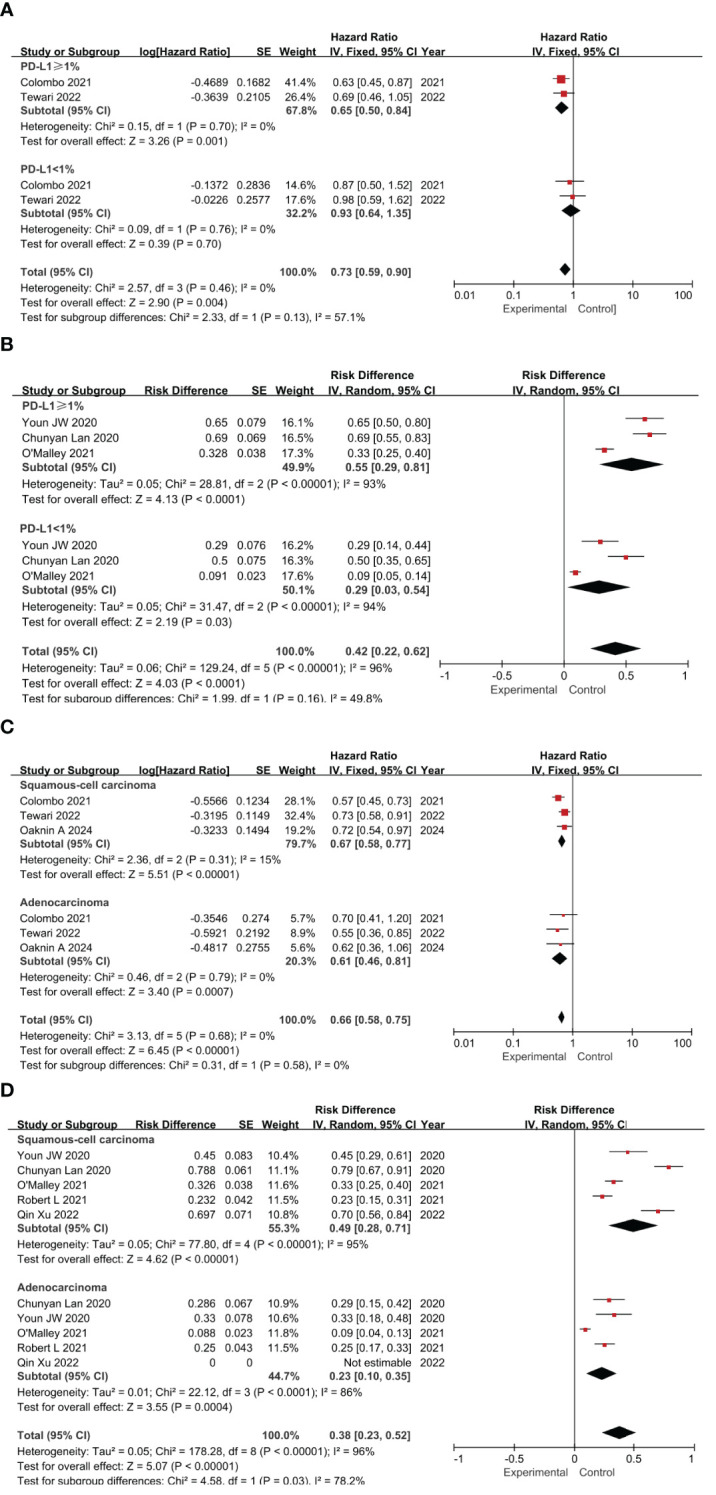
Forest plots of subgroup analysis categorized by clinical characteristics: PD-L1 Expression Impact on OS in RCTs **(A)** and ORR in Single-Arm Studies **(B)**; Histological Type Impact on OS in RCTs **(C)** and ORR in Single-Arm Studies **(D)**.

Patients were stratified by PD-L1 combined positive score (CPS) thresholds (≥1 vs. <1) to assess treatment effects. In RCTs, PD-L1 ≥1 was associated with significantly OS versus control (HR=0.65, 95% CI: 0.50–0.84, *P*=0.001), while the CPS < 1% subgroup showed a non-significant effect (HR=0.93, 95% CI: 0.64–1.35, *P*=0.70). The test for subgroup differences yielded a P-value of 0.13, indicating no significant difference in effect sizes between the two subgroups. The pooled analysis confirmed the superior efficacy of the intervention over controls (HR=0.73, 95% CI: 0.59–0.90, *P*=0.004) (**Figure 4A**). In single-arm studies, PD-L1 ≥1% had a higher risk difference (RD=0.55) than CPS <1% (RD=0.29), though subgroup differences were not significant (*P*=0.16) (**Figure 4B**). Subgroup analysis by histology revealed significant OS benefits for both squamous cell carcinoma (HR=0.67, *P*<0.00001) and adenocarcinoma (HR=0.61, *P*=0.0007), with consistent results (I²=0%) (**Figure 4C**). Single-agent ICIs showed higher efficacy in squamous cell carcinoma (RD=0.49) than adenocarcinoma (RD=0.23), with significant subgroup differences (*P*=0.03) (**Figure 4D**). Overall, ICIs demonstrated superior efficacy, with treatment effects varying by PD-L1 expression and histologic type.

### Sensitivity analysis and risk of bias

**Figure 5:**
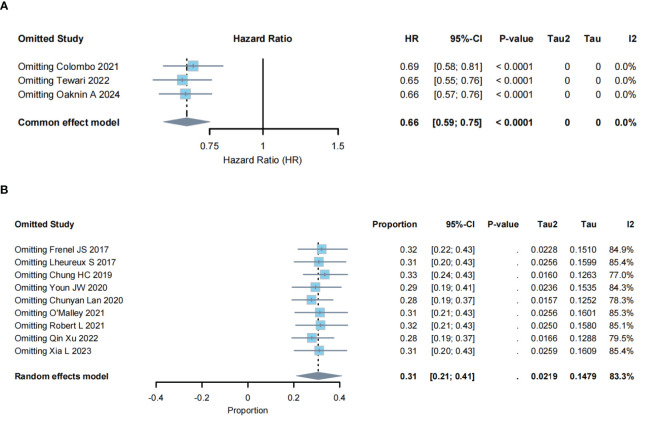
Sensitivity analysis of included studies: **(A)** RCTS; **(B)** single-arm studies.

Sensitivity analysis was performed to evaluate the impact of individual studies on the pooled results. For OS in RCTs, the HR remained stable (range: 0.65–0.69), with substantial overlap in 95% confidence intervals and minimal heterogeneity (I²=0%), indicating robust and reliable results (**Figure 5A**). For ORR in single-arm studies, the response proportion remained stable (range: 0.28–0.33), with substantial overlap in 95% confidence intervals. However, heterogeneity remained high (I²=77%–85.4%), and Tau² values showed minor fluctuations (**Figure 5B**). No single study disproportionately influenced the ORR estimate, indicating robust pooled results despite high heterogeneity. The 95% CI from the remaining studies provided valuable insights after excluding most publications. Risk of bias was assessed using ROB-2 and ROBINS-I (**Supplementary Materials Appendix B**). Based on ROB-2, three RCTs were classified as low risk (23–25). Using ROBINS-I, two single-arm studies were classified as low risk (26, 30), and nine as moderate risk (27–29, 31–34). This stratification provided insights into the influence of study quality on the findings.

## Discussion

### Summary of systematic review results

CC remains one of the most prevalent diseases affecting women globally, but treatment alternatives are limited when the disease progresses to an advanced stage or recurs. In terms of vaccination coverage, it is still inadequate, and only those who were born after the early 1990s have had access to it before engaging in sexual activity (43). Considering the grim prognosis for patients with metastatic or recurrent cancer, there is an urgent clinical demand for effective therapeutic options that warrant additional investigation. Immunotherapy stands as a promising treatment modality for such patients. The hypothesis suggests that CC, primarily caused by chronic HPV infection, may be especially susceptible to treatment with ICIs. With the explosion of novel therapeutic agents and combination regimens in recent years, immunotherapy has steadily emerged as a focal point of advanced CC research, numerous clinical trials are actively underway to evaluate the potential impact of ICIs in this malignancy (44, 45).

Encouraging advancements in efficacy and safety have been observed in our systematic review of patients diagnosed with advanced CC receiving ICIs. Meanwhile, whether patients with CC can benefit from ICI treatments and the selection of specific treatment protocols, suitable populations need to be studied in depth. Due to the wide array of trial designs, therapeutic agents and combinations, prior treatment regimens, and patient selection criteria, the outcomes of these studies exhibit considerable variability. Further confirmation data from randomized studies is awaited.

In conclusion, the Food and Drug Administration (FDA) approved pembrolizumab as the sole agent for patients with PD-L1-positive metastatic/recurrent CC, based on the promising results of phase I/II trials. Notably, pembrolizumab remains the only medication with published phase III trial data demonstrating an extended OS of approximately eight months and has recently been licensed for use in patients with advanced CC (46, 47). Furthermore, 2023 ASCO released 39.1-month follow-up results from the KEYNOTE-826 trial, with updated data continuing to show that pembrolizumab plus chemotherapy, with or without bevacizumab, significantly improved overall survival in patients with persistent, recurrent, or metastatic cervical cancer. Median OS was 28.6 months (PD-L1 CPS≥1), 26.4 months (all-comer), and 29.6 months (CPS≥10). Grade ≥3 adverse events occurred in 82.4% of the pembrolizumab group and 75.4% of the placebo group (47, 48). In the BEATcc trial, 410 patients with metastatic, persistent, or recurrent cervical cancer were randomly assigned to receive standard therapy with or without atezolizumab. The result showed that addition of atezolizumab to standard therapy significantly improved PFS and OS, suggesting a new first-line treatment option for advanced cervical cancer (25). In parallel, EMPOWER-Cervical 1 trial showed the mOS was 12.0 months in the cemiplimab group versus 8.5 months in the chemotherapy group, indicating that cemiplimab offers significant survival benefits and a favorable safety profile in patients with recurrent cervical cancer (24).

Our comprehensive analysis, encompassing both RCTs and single-arm studies, provides valuable insights into the efficacy and safety profiles of ICIs in this clinical context. In light of our systematic review, we underscore the significant efficacy of ICIs in improving OS and PFS in patients with advanced cervical cancer (CC). The pooled HR for OS (0.66) and PFS (0.67) indicate a substantial reduction in the risk of death and disease progression, respectively, consistent with results from pivotal trials such as KEYNOTE-826 and BEATcc. Notably, the ORR was higher in ICI-treated patients, further supporting their therapeutic potential. Safety considerations reveal ICIs exhibit a distinct toxicity profile compared to chemotherapy or anti-angiogenic agents. The analysis showed that the safety profile of ICIs is manageable, with no significant increase in severe adverse events (≥G3) compared to control groups in RCTs. However, immune-related AEs (irAEs) require specialized management, single-arm studies reported a higher incidence of adverse events, underscoring the need for careful monitoring and patient selection. Managing irAEs from ICIs requires a tailored approach. Mild irAEs (Grade 1-2) typically necessitate treatment interruption and symptom management, while severe irAEs (Grade ≥3) require prompt intervention with corticosteroids or other immunosuppressive agents. Single-arm studies have reported a higher incidence of adverse events, highlighting the importance of careful patient selection and monitoring. Future research should focus on identifying predictive biomarkers to optimize patient selection, minimize adverse events, and enhance the efficacy and safety of ICIs.

Subgroup analyses revealed that PD-L1 expression and histologic type significantly influence treatment outcomes, with PD-L1 CPS ≥1 and squamous cell carcinoma associated with greater benefits. These findings emphasize the importance of biomarker-driven strategies to optimize ICI use in advanced CC. Currently, numerous trials are underway to optimize immunotherapy for advanced cervical cancer. Studies like NCT04300647, DUBHE-C-204, and QL1706-301 are exploring novel combinations and innovative approaches to enhance treatment efficacy (48, 49). Beyond these, researchers are investigating cancer vaccines, genome editing tools, engineered T cells, herbal extracts, interleukins, and cytokines to modulate the immune response (50, 51). The significant heterogeneity in current studies highlights the need for further exploration. The ultimate goal is to develop personalized treatment regimens that prolong survival and improve quality of life by tailoring therapies to each patient’s unique tumor and immune profile.

### Limitations of the systematic review

This systematic review has several limitations that warrant consideration. First and foremost, it is noteworthy that most of the included articles were non-comparable studies, and a significant proportion had small sample sizes, with inherent limitations in methodological rigor that may compromise the reliability of comparative outcome assessments. Second, there were variations in the immune checkpoint inhibitors utilized in each study, which unavoidably led to bias. Third, critical biomarker data required for subgroup interpretation were frequently absent: HPV status, microsatellite instability (MSI) testing, and comprehensive tumor mutational burden (TMB) data were unavailable. This missing biomarker dimension fundamentally constrains our ability to identify molecular predictors of ICI response. Finally, the predominantly descriptive nature of safety reporting and short median follow-up duration limit longitudinal assessment of both survival outcomes and late-onset toxicities.

As such, the conclusions about the effectiveness and safety of ICIs in advanced CC that can be made from our analysis are merely descriptive. We posit that the conduct of more randomized controlled trials, coupled with an extended follow-up period, would elucidate the precise impact of ICIs on the survival outcomes of patients with advanced cancer. Additionally, further research is warranted to identify appropriate patient selection criteria and develop a personalized treatment approach. Future trials should prioritize (1) standardized MSI/HPV/TMB reporting, (2) head-to-head comparison of ICI sequencing strategies, and (3) longitudinal quality-of-life metrics to inform value-based treatment algorithms.

## Conclusions

Immunotherapy holds significant promise as a treatment modality for patients with advanced cancer, offering the potential for long-lasting responses and controllable toxicity. Current trials assess ICIs in combination with RT, CRT, or cancer vaccines. However, there is a lack of high-level research data. In clinical practice, careful patient selection and monitoring are crucial due to the potential for irAEs associated with ICIs. These irAEs can affect various organs and require specialized management strategies, including corticosteroids or other immunosuppressive agents for severe cases. Patient tolerance and general health should be considered when adjusting ICIs dosage, especially in fragile, elderly, or frail patients. Further research is needed to determine the best patient population, treatment approach, and administration time. Longer observation periods may confirm results and further investigation is needed for patient pooling and tailored strategies.

## Data Availability

The datasets presented in this study can be found in online repositories. The names of the repository/repositories and accession number(s) can be found in the article/**Supplementary Material**.
