# Supplementary material for: Effectiveness and safety of ICIs for the treatment of advanced CC: a systematic review and meta-analysis
Source: Front Immunol. 2025 Mar 11;16:1542850. doi: 10.3389/fimmu.2025.1542850 (PMC11933070; doi:10.3389/fimmu.2025.1542850)

*Systematic Review*

**Effectiveness and safety of ICIs for the treatment of** **advanced cervical cancer：A systematic review and meta-analysis**

Nurbia Ibibulla^1^ , Pengfei Lu^1^ , Yiliyaer Nuerrula^1^ , Xueqin Hu^1^ , Mulati Aihemaiti^1^ , Yubo Wang^2^* , Hua Zhang^1^*

**Supplementary Appendix**

Appendix A. Search Strategy for Databases

Appendix B. Risk of bias assessment

**Appendix A. Search Strategy for Databases**

1. PubMed

Search string

Subject heading: **Uterine Cervical Neoplasms;** **Immune Checkpoint Inhibitors**

**(("****Uterine Cervical Neoplasms"[Mesh]) OR (((((((((((((((((Uterine Cervical Neoplasm[Title/Abstract]) OR (Cervical Neoplasm*, Uterine[Title/Abstract])) OR (Neoplasm*, Uterine Cervical[Title/Abstract])) OR (Neoplasm*, Cervical[Title/Abstract])) OR (Cervical Neoplasm*[Title/Abstract])) OR (Neoplasm*, Cervix[Title/Abstract])) OR (Cervix Neoplasm*[Title/Abstract])) OR (Cancer of the Uterine Cervix[Title/Abstract])) OR (Cancer of the Cervix[Title/Abstract])) OR (Cervical Cancer*[Title/Abstract])) OR (Cancer*, Cervical[Title/Abstract])) OR (Cancer*, Uterine Cervical[Title/Abstract])) OR (Cervical Cancer*, Uterine[Title/Abstract])) OR (Uterine Cervical Cancer*[Title/Abstract])) OR (Cancer of Cervix[Title/Abstract])) OR (Cervix Cancer*[Title/Abstract])) OR (Cancer*, Cervix[Title/Abstract]))) AND (("Immune Checkpoint Inhibitors"[Mesh]) OR (((((((((((((((((((((((((((((((((((((((ICIs[Title/Abstract]) OR (Immune Checkpoint Inhibitor[Title/Abstract])) OR (Checkpoint Inhibitor*, Immune[Title/Abstract])) OR (****Immune Checkpoint Blocker*[Title/Abstract])) OR (Checkpoint Blocker*, Immune[Title/Abstract])) OR (****Immune Checkpoint Blockade[Title/Abstract])) OR (Checkpoint Blockade ,Immune[Title/Abstract])) OR (Immune Checkpoint Inhibition[Title/Abstract])) OR (Checkpoint inhibition ,immune[Title/Abstract])) OR (****PD-L1 Inhibitor*[Title/Abstract])) OR (****PD-1 Inhibitor*[Title/Abstract])) OR (PD L1 Inhibitor*[Title/Abstract])) OR (PD 1 Inhibitor*[Title/Abstract])) OR (Inhibitor*, PD-1[Title/Abstract])) OR (Programmed Death-Ligand 1 Inhibitor*[Title/Abstract])) OR (Programmed Death Ligand 1 Inhibitor*[Title/Abstract])) OR (Programmed Cell Death Protein 1 Inhibitor*[Title/Abstract])) OR (****PD-1-PD-L1 Blockade[Title/Abstract])) OR (Blockade,PD-1-PD-L1[Title/Abstract])) OR (PD 1 PD L1 Blockade[Title/Abstract])) OR (CTLA-4 Inhibitor*[Title/Abstract])) OR (CTLA 4 Inhibitor*[Title/Abstract])) OR (Cytotoxic T Lymphocyte Associated Protein 4 Inhibitor*[Title/Abstract])) OR (Cytotoxic T -Lymphocyte-Associated Protein 4 Inhibitor*[Title/Abstract])) OR (Pembrolizumab[Title/Abstract])) OR (Nivolumab[Title/Abstract])) OR (****Atezolizumab[Title/Abstract])) OR (Durvalumab[Title/Abstract])) OR (Cemiplimab[Title/Abstract])) OR (Camrelizumab[Title/Abstract])) OR (Sintilimab[Title/Abstract])) OR (Tislelizumab[Title/Abstract])) OR (Toripalimab[Title/Abstract])) OR (Avelumab[Title/Abstract])) OR (Tremelimumab[Title/Abstract])) OR (****Ipilimumab[Title/Abstract])) OR (Dostarlimab[Title/Abstract])) OR (Balstilimab[Title/Abstract])) OR (Camrelizumab[Title/Abstract])))**

**Search returned 313 articles;**

2.Embase

Search string

1. **'uterine cervix tumor'/exp**
2. **'cervical neoplasia':ab,ti OR 'cervical neoplasm':ab,ti OR 'cervical tumor':ab,ti OR 'cervical tumorigenesis':ab,ti OR 'cervical tumour':ab,ti OR '****cervix neoplasia':ab,ti OR '****cervix neoplasm':ab,ti OR 'cervix neoplasms':ab,ti OR 'cervix tumor':ab,ti OR 'cervix tumorigenesis':ab,ti OR 'cervix tumour':ab,ti OR 'cervix uteri tumor':ab,ti OR 'neoplasia of the cervix':ab,ti OR '****neoplasm of the cervix':ab,ti OR 'neoplastic cervical':ab,ti OR '****neoplastic cervix':ab,ti OR 'tumor of the cervix':ab,ti OR '****tumor of the uterine cervix':ab,ti OR 'tumour of the cervix':ab,ti OR 'tumour of the uterine cervix':ab,ti OR 'uterine cervical neoplasia':ab,ti OR 'uterine cervical neoplasm':ab,ti OR 'uterine** **cervical neoplasms':ab,ti OR 'uterine cervical tumor':ab,ti OR 'uterine cervix neoplasia':ab,ti OR 'uterine cervix neoplasm':ab,ti OR 'uterine cervix tumour':ab,ti OR 'uterine cervix tumor':ab,ti**
3. **#1 OR #2**
4. **'****immune checkpoint inhibitor'/exp**
5. **'immune checkpoint blocker':ab,ti OR 'immune checkpoint inhibitor':ab,ti OR 'pd 1 inhibitor':ab,ti OR 'immune checkpoint blockade':ab,ti OR 'immune checkpoint inhibition':ab,ti OR 'pd l1 inhibitor':ab,ti OR 'programmed death ligand 1 inhibitor':ab,ti OR 'programmed cell death protein 1 inhibitor':ab,ti OR pembrolizumab:ab,ti OR nivolumab:ab,ti OR atezolizumab:ab,ti OR durvalumab:ab,ti OR cemiplimab:ab,ti OR ipilimumab:ab,ti OR camrelizumab:ab,ti**
6. **#4 OR #5**
7. **#3 AND #6 /py**

**Search returned 861 articles;**

3.Medline

**TX ( Uterine Cervical Neoplasms OR Uterine Cervical Neoplasm OR Cervical Neoplasm, Uterine OR Neoplasm , Uterine Cervical OR Neoplasms, Cervical OR Cervical Neoplasm* OR Neoplasms, Cervix OR Cervix Neoplasm* OR Cancer of the Uterine Cervix OR Cancer of Cervix OR cervical cancer* OR Cancer, Cervical OR cervical carcinoma OR Uterine Cervical Cancer* OR Cancer, Uterine Cervical OR cervical cancer,Uterine OR Cervix Cancer OR Cancer of the Cervix OR carcinoma of cervix OR Cancer, Cervix ) AND TX ( Immune Checkpoint Inhibitors OR ICIs OR Immune Checkpoint Inhibitor OR Checkpoint Inhibitor*, Immune OR Immune Checkpoint Blocker* OR Checkpoint Blocker*, Immune OR Immune Checkpoint Blockade OR Checkpoint Blockade, Immune OR Immune Checkpoint Inhibition OR Checkpoint Inhibition, immune OR PD-L1 Inhibitor* OR PD-1 Inhibitor* OR PD L1 Inhibitor* OR PD 1 Inhibitor* OR Inhibitor*, PD-1 OR Programmed Death-Ligand 1 Inhibitor* OR Programmed Death Ligand 1 Inhibitor* OR Programmed Cell Death Protein 1 Inhibitor* OR PD-1-PD-L1 Blockade OR Blockade, PD-1-PD-L1 OR PD 1 PD L1 Blockade OR CTLA-4 Inhibitor* OR CTLA 4 Inhibitor* OR Cytotoxic T Lymphocyte Associated Protein 4 Inhibitor* OR Cytotoxic T -Lymphocyte-Associated Protein 4 Inhibitor* OR Pembrolizumab OR Nivolumab OR Atezolizumab OR Camrelizumab OR Sintilimab OR Tislelizumab OR Toripalimab OR Ipilimumab OR Dostarlimab OR Balstilimab OR Camrelizumab OR Durvalumab OR Cemiplimab OR Avelumab OR Tremelimumab ) Full Text;**

Search returned 278 articles

4. Web of Science

Search string

**TS=(****Uterine Cervical Neoplasms OR Uterine Cervical Neoplasm OR Cervical Neoplasm*, Uterine OR** **Neoplasm*, Cervical OR Cervical Neoplasm* OR Neoplasm*, Cervix OR Cervix Neoplasm* OR Cancer of the Uterine Cervix OR Cancer of Cervix OR cervical cancer* OR Cancer*, Cervical OR cervical carcinoma OR Uterine Cervical Cancer* OR Cancer*, Uterine Cervical OR cervical cancer*, Uterine OR** **Cervix Cancer* OR Cancer of the Cervix OR carcinoma of cervix OR Cancer*, Cervix) AND TS=(** **Immune Checkpoint Inhibitors OR ICIs OR Immune Checkpoint Inhibitor OR Checkpoint Inhibitor*, Immune OR Immune Checkpoint Blocker* OR Checkpoint Blocker*, Immune OR Immune Checkpoint Blockade OR Checkpoint Blockade, Immune OR** **Immune Checkpoint Inhibition OR Checkpoint Inhibition, immune OR PD-L1 Inhibitor* OR PD-1 Inhibitor* OR PD L1 Inhibitor* OR PD 1 Inhibitor* OR Inhibitor*, PD-1 OR** **Programmed Death-Ligand 1 Inhibitor* OR Programmed Death Ligand 1 Inhibitor* OR Programmed Cell Death Protein 1 Inhibitor* OR PD-1-PD-L1 Blockade OR Blockade, PD-1-PD-L1 OR PD 1 PD L1 Blockade OR CTLA-4 Inhibitor* OR CTLA 4 Inhibitor* OR Cytotoxic T Lymphocyte Associated Protein 4 Inhibitor* OR Cytotoxic T -Lymphocyte-Associated Protein 4 Inhibitor* OR Pembrolizumab OR Nivolumab OR Atezolizumab OR Camrelizumab OR Sintilimab OR** **Tislelizumab OR** **Toripalimab OR** **Ipilimumab OR Dostarlimab OR** **Balstilimab OR** **Camrelizumab OR** **Durvalumab OR Cemiplimab OR Avelumab OR** **Tremelimumab)**

**Search returned 722 articles;**

5. Cochrane

Search string

Subject heading: Uterine Cervical Neoplasms; Immune Checkpoint Inhibitors

1. **MeSH descriptor: [Immune Checkpoint Inhibitors] explode TI trees**
2. **PD L1 Inhibitors or PD L1 Inhibitor or Programmed Death-Ligand 1 Inhibitors or Programmed Death Ligand 1 Inhibitors or PD 1 PD L1 Blockade or Immune Checkpoint Blockers or Immune Checkpoint Inhibitor or Immune Checkpoint Inhibitors or CTLA-4 Inhibitor or Cytotoxic T-Lymphocyte-Associated Protein 4 Inhibitor or Cytotoxic T-Lymphocyte-Associated Protein 4 Inhibitors or CTLA-4 Inhibitors or Cytotoxic T Lymphocyte Associated Protein 4 Inhibitor or Cytotoxic T Lymphocyte Associated Protein 4 Inhibitors or PD 1 Inhibitors or PD 1 Inhibitor or Programmed Cell Death Protein 1 Inhibitor or Programmed Cell Death Protein 1 Inhibitors or Immune Checkpoint Inhibition or Immune Checkpoint Blockade**
3. **#1 and #2**
4. **MeSH descriptor: [Uterine Cervical Neoplasms] explode TI trees**
5. **Neoplasm** **or Uterine Cervical or Cervical Neoplasm, Uterine** **or Uterine Cervical Neoplasm or Cervix Neoplasms or Neoplasms, Cervical or Cervix Neoplasm or Neoplasm, Cervix or Neoplasms, Cervix or Cervical Neoplasms** **or Cervical Neoplasm or Cervical Cancers or Cancer of Cervix or Uterine Cervical Cancer or Cervix Cancer or Cancer, Cervix or Cancer, Uterine Cervical or Uterine Cervical Cancers or Cancer of the Uterine Cervix or Cervical Cancer or Cervical Cancer, Uterine or Cancer, Cervical or Cancer of the Cervix**
6. **#4 or #5**
7. **#3 and #6**

**Search returned 476 article**

**Appendix B. Risk of bias assessment**

1. RoB-2 Assessment Form


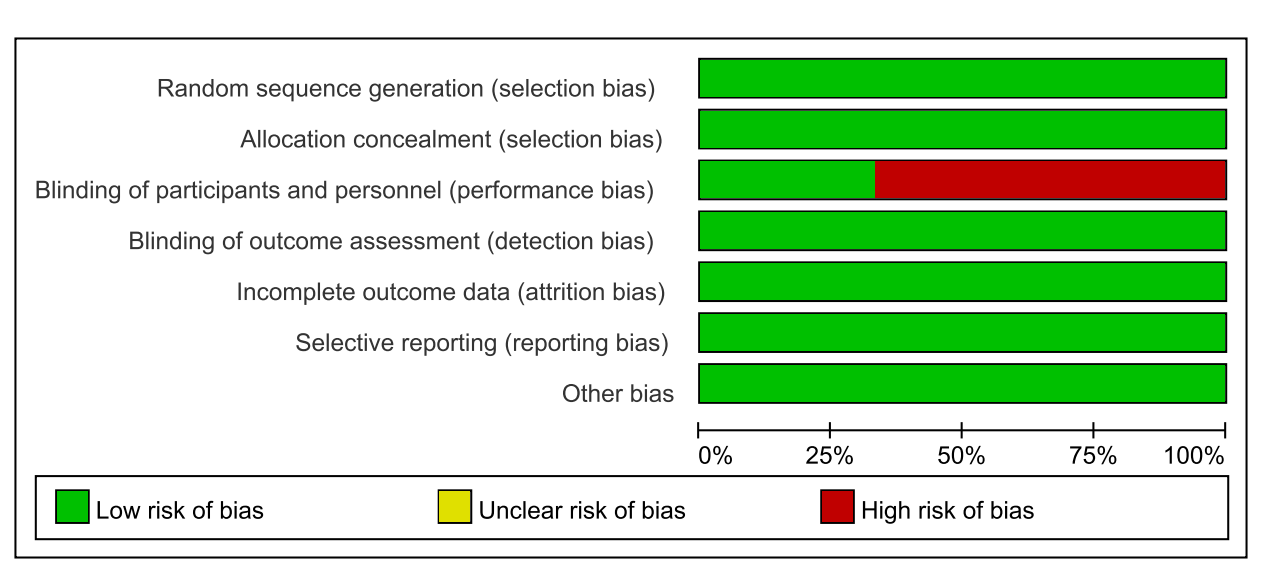


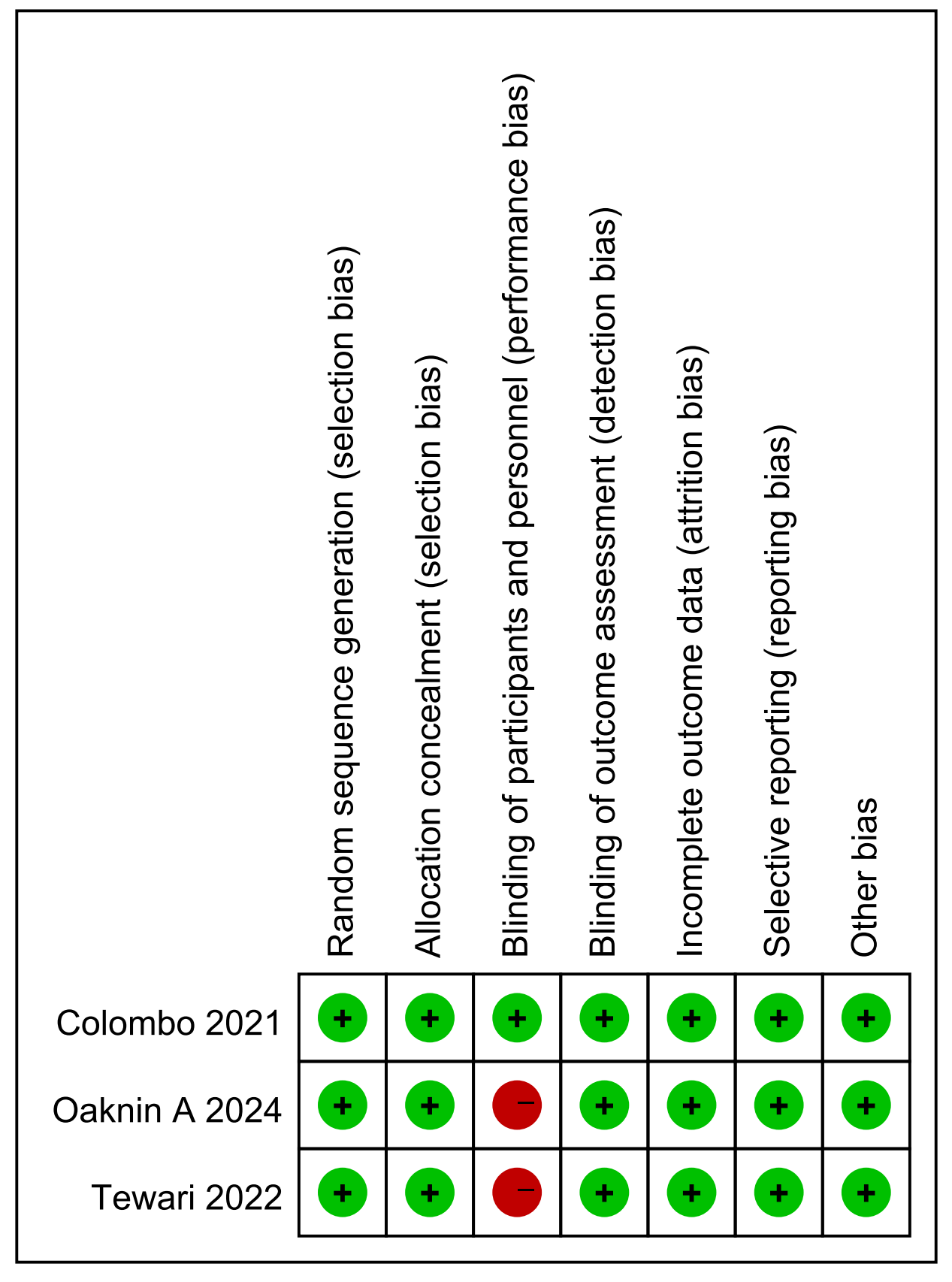


1. Risk of bias for the 11 included publications (single arm studies), based on the ROBINS-I tool (low, moderate, serious, critical)


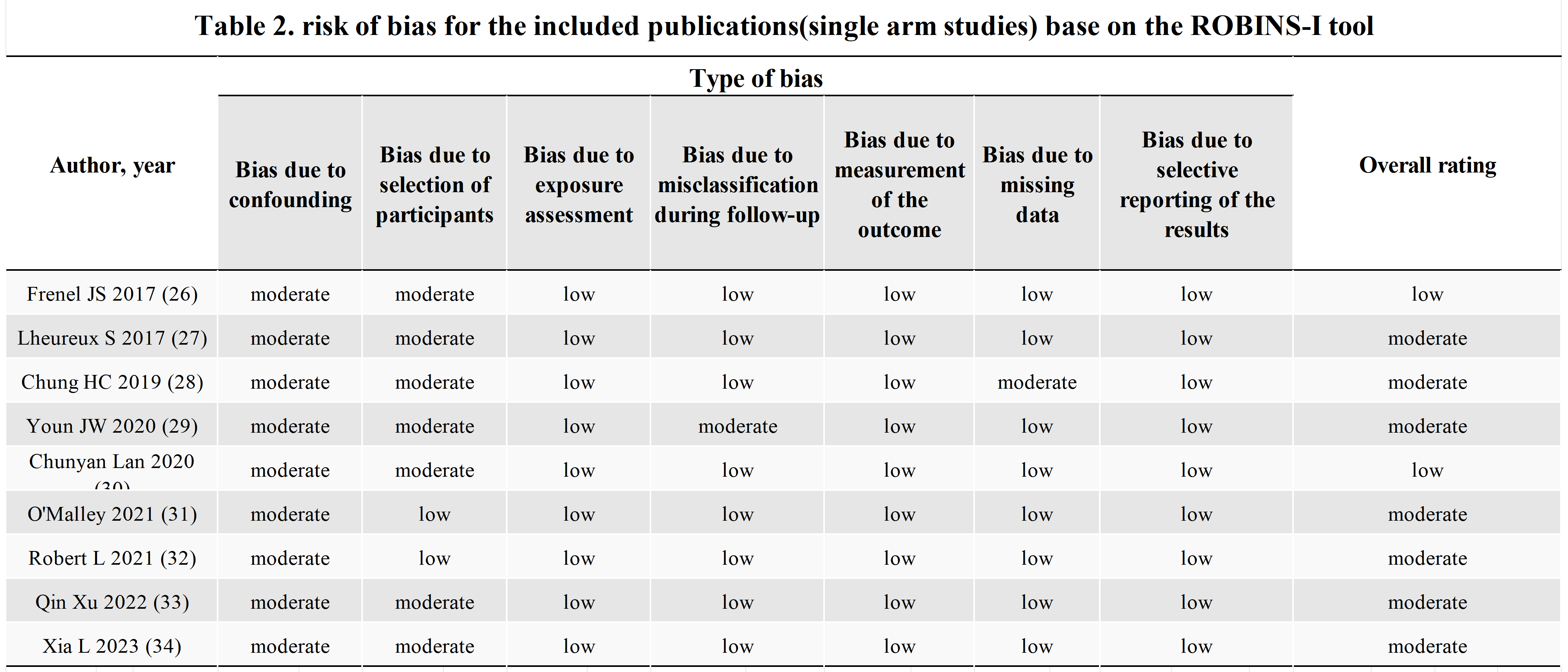

Supplement: Supplementary file 1 [file DataSheet1.zip › supporting information/Supplementary materials.doc]
